# Factors associated with postoperative signs and symptoms in teeth with periapical lesion: a longitudinal study

**DOI:** 10.1590/0103-6440202205040

**Published:** 2022-12-05

**Authors:** Erlange Andrade Borges Silva, Ludmila Silva Guimarães, Fernanda Garcias Hespanhol, Caio Luiz Bitencourt Reis, Lívia Azeredo Alves Antunes, Leonardo Santos Antunes

**Affiliations:** 1 Postgraduate Program, School of Dentistry, Fluminense Federal University, Niterói, RJ, Brazil.; 2 Department of Pediatric Dentistry, School of Dentistry of Ribeirão Preto, University of São Paulo.; 3 Department of Specific Formation, School of Dentistry, Fluminense Federal University, Nova Friburgo, RJ, Brazil.; 4 Postgraduate Program in Dentistry of Health Institute of Nova Friburgo, Fluminense Federal University, Nova Friburgo, RJ, Brazil.

**Keywords:** periodontitis, hypertension, blood pressure, bacteria, real-time polymerase chain reaction

## Abstract

This study aimed to evaluate the association of the variables age, gender, arch position, tooth length, root canal amplitude, and periapical lesion size with the occurrence of postoperative signs and symptoms (pain, tenderness, and edema) and the use of postoperative analgesics following root canal treatment with foraminal enlargement in single-rooted teeth with apical periodontitis. This prospective longitudinal study included 105 patients requiring root canal treatment of maxillary or mandibular single-rooted teeth with periapical lesion. After root canal treatment in a single session, pain intensity and tenderness were recorded daily for 7 days and on days 14 and 30. Edema was evaluated by two independent evaluators within 48 h, 72 h, and 7 days after treatment. Ordinal and logistic regressions were performed (p < 0.05). Female gender (beta = 1.02; p < 0.01), mandibular teeth (beta = 25.50; p < 0.01), medium root canal amplitude (beta = 0.93; p = 0.03), and edema (beta = 1.88; p < 0.01) were associated with increased postoperative pain and tenderness, while the use of analgesics (beta = -1.82; p < 0.01) and time in days (beta = -0.23; p < 0.01) were associated with a decrease in these signs and symptoms. Edema was considered a risk factor for analgesic requirement (Odds Ratio [OR] = 61.46; p < 0.01). Factors such as gender, arch position, and root canal amplitude were associated with postoperative signs and symptoms. The use of analgesics was more required in edema and was associated with decreased pain.

## Resumo

Este estudo teve como objetivo avaliar a associação das variáveis idade, sexo, posição no arco, comprimento do dente, amplitude do canal radicular e tamanho da lesão periapical com a ocorrência de sinais e sintomas pós-operatórios (dor, dor ao toque e edema) e o uso de analgésicos após o tratamento endodôntico com alargamento foraminal em dentes uniradiculares com lesão periapical. Este estudo longitudinal prospectivo incluiu 105 pacientes que necessitavam de tratamento endodôntico em dentes uniradiculares superiores ou inferiores com lesão periapical. Após o tratamento do canal radicular em uma sessão, a intensidade da dor e a dor ao toque foram registradas diariamente por 7 dias e nos dias 14 e 30. O edema foi avaliado por dois avaliadores independentes dentro de 48 h, 72 h e 7 dias após o tratamento. Foram realizadas regressões ordinal e logística, e a significância estatística foi fixada em um valor de p < 0,05. Gênero feminino (beta = 1,02; p < 0.01), dentes inferiores (beta = 25,50; p < 0.01), amplitude média do canal radicular (beta = 0,93; p = 0,03) e edema (beta = 1,88; p < 0.01) foram associados ao aumento da dor e dor ao toque pós-operatória, enquanto o uso de analgésicos (beta = -1,82; p < 0.01) e o tempo em dias (beta = -0,23; p < 0.01) foram associados à diminuição desses sinais e sintomas. O edema foi considerado fator de risco para necessidade de analgésico (Odds Ratio [OR] = 61,46; p < 0.01). Fatores como sexo, posição do arco e amplitude do canal radicular foram associados aos sinais e sintomas pós-operatórios. O uso de analgésicos foi mais necessário no edema e foi associado à diminuição da dor.

## Introduction

Postoperative symptoms can occur within a few hours or days after root canal treatment and comprise mild to acute exacerbations that mainly affect necrotic teeth with periapical lesion ^1^ ^,^ ^2^. Although this definition varies widely among authors, postoperative signs and symptoms are well-known complications that disturb both patients and dentists ^2^. Its incidence has been reported to range from 1.58% to 58% ^1^ ^,^ ^2^ ^,^ ^3^ ^,^ ^4^ ^,^ ^5^ ^,^ ^6^.

The causative factors of postoperative pain and edema include mechanical, chemical, and/or microbial injury to the pulp or periradicular tissues ^4^. In addition, some studies have correlated the postoperative signs and symptoms after root canal treatment with many variables, such as different tooth group ^1^ ^,^ ^2^ ^,^ ^3^ ^,^ ^5^ ^,^ ^7^ ^,^ ^8^ ^,^ ^9^ ^,^ ^10^ ^,^ ^11^ ^,^ ^12^ ^,^ ^13^, arch position ^1^ ^,^ ^2^ ^,^ ^3^ ^,^ ^5^ ^,^ ^7^ ^,^ ^8^ ^,^ ^11^ ^,^ ^12^ ^,^ ^14^ ^,^ ^15^, gender ^1^ ^,^ ^2^ ^,^ ^3^ ^,^ ^5^ ^,^ ^6^ ^,^ ^7^ ^,^ ^8^ ^,^ ^9^ ^,^ ^10^ ^,^ ^12^ ^,12,^ ^14^ ^,^ ^15^ ^,^ ^16^, age ^1^ ^,^ ^2^ ^,^ ^3^ ^,^ ^5^ ^,^ ^7^ ^,^ ^8^ ^,^ ^9^ ^,^ ^12^ ^,^ ^14^ ^,^ ^15^ ^,^ ^16^, and the size ^7^ ^,^ ^8^ ^,^ ^10^ or presence of periapical lesion ^1^ ^,^ ^2^ ^,^ ^3^ ^,^ ^4^ ^,^ ^5^ ^,^ ^12^ ^,^ ^14^ ^,^ ^16^.

Although several studies ^1^ ^,^ ^2^ ^,^ ^3^ ^,^ ^4^ ^,^ ^5^ ^,^ ^6^ ^,^ ^7^ ^,^ ^8^ ^,^ ^9^ ^,^ ^10^ ^,^ ^11^ ^,^ ^12^ ^,^ ^13^ ^,^ ^14^ ^,^ ^15^ ^,^ ^16^ have investigated the role of these variables in postoperative signs and symptoms, there is a lack of conclusive data in the literature, especially regarding tooth length, root canal amplitude, and periapical lesion size. In addition, foraminal enlargement was not carried out in these studies, and most of them ^2^ ^,^ ^3^ ^,^ ^4^ ^,^ ^6^ ^,^ ^7^ ^,^ ^9^ ^,^ ^11^ ^,^ ^12^ ^,^ ^16^ performed the root canal instrumentation manually, leading to greater extrusion of debris ^17^ and consequently greater postoperative symptoms ^4^. It is believed that the proper identification of these factors can help improve patient management and outcomes ^6^.

Therefore, this prospective longitudinal study aimed to evaluate the relationship between age, gender, arch position, tooth length, root canal amplitude, and periapical lesion size with the occurrence of postoperative signs and symptoms and use of analgesics, following root canal treatment with foraminal enlargement in single-rooted teeth with periapical lesion in a single session. The null hypothesis was that there is no relationship between these factors and the occurrence of postoperative signs and symptoms and analgesic requirements.

## Material and methods

### Study Design and Ethical Issues

This prospective longitudinal study was approved by the local ethics committee (no. 2.353.996), and was reported following the Guidelines for reporting non-randomized studies ^18^. An informed consent form was obtained from all volunteers to participate in the study. In addition, all the risks and benefits of the treatment were also conveyed to the participants.

### Patient Selection

The study population comprised patients over 18 years of age who presented to the Faculty of Dentistry of the Fluminense Federal University/Health Institute of Nova Friburgo, Rio de Janeiro, Brazil, for the evaluation and management of deep carious lesions, between March 2019 and February 2020.

The sample size was determined using the OpenEpi calculator (https://www.openepi.com/Menu/OE_Menu.htm). The sample was calculated by estimating the prevalence of severe postoperative pain on the first day after root canal treatment with foraminal enlargement of necrotic teeth, which was reported to be 34% ^19^, with a 10% error and 95% confidence interval. To compensate for a possible cluster effect, the sample size was increased by 20% (design effect = 1.2), which yielded a total sample size of 104 participants.

Patients included in the study required at least one root canal treatment on the maxillary or mandibular single-rooted teeth with periapical lesion without preoperative pain or edema. The exclusion criteria for the study were as follows: 1) patients with systemic disorders; 2) allergy to ibuprofen; 3) used antibiotics in the last 30 days ^19^; 4) inability to determine the foramen patency, with a wide apical foramen, allergy to sodium hypochlorite (NaOCl), cases of endodontic retreatment, and vital teeth.

### Treatment Protocol

A single specialist (E.A.B.S), with 35 years of experience, performed the clinical procedures in a single session using a standardized protocol. Digital periapical radiography (Kodak, Rochester, New York, USA) was performed in a standardized form to aid in diagnosis and follow-up. Dental biofilms were removed by professional oral prophylaxis, and the patients were instructed to rinse with 15 mL of 0.12% chlorhexidine gluconate for 1 min (Periogard alcohol-free; Colgate Palmolive Company, Cambridge, Ohio, USA). The diagnosis of pulp necrosis was determined by sensitivity tests to cold with Endo Ice (Coltene/Whaledent Inc, Cuyahoga Falls, Ohio, USA) and hot with a heated gutta-percha stick (Dentsply Sirona, York, Pennsylvania, USA). Patients were anesthetized with 2% lidocaine with 1:100,000 epinephrine (Alphacaine; DFL Indústria e Comércio Ltda, Taquara, Rio de Janeiro, Brazil), and endodontic access was performed with a sterile diamond bur (KG Sorensen, Cotia, São Paulo, Brazil), at high speed. Rubber dam was placed and disinfected with 2.5% NaOCl (Fórmula & Ação, São Paulo, Brazil). The working length (WL) was measured with a size 15 K-file (Dentsply Sirona), and the patency was maintained with a size 10 K-file (Dentsply Sirona). Odontometry determination was performed using the apical locator RomiApex A-15 (Romidan, Kiryat Ono, Israel), and WL was established at the “00” mark.

Based on the initial radiographs and the anatomical diameter of the root canal determined by initial exploration, up to the WL, with sizes 10, 15, 20, 25, and 30 K-files (Dentsply Sirona), under constant irrigation, 40 or 50 Reciproc files (VDW, Munich, Germany), coupled to the Silver engine (VDW, München, Germany), were selected for instrumentation. In cases where a size 30 K-file did not move passively to the WL, R40 was selected, and these cases were classified as medium. In cases where a size 30 K-file passed passively to the WL, R50 was selected, and these cases were classified as large, according to the manufacturer's protocol. The instruments were introduced with linear back-and-forth movements with an amplitude of 2(3 mm, with slight apical pressure. The files were discarded after a single use.

Each tooth was irrigated with the same volume of irrigant, 15 mL of 2.5% NaOCl (Fórmula & Ação), using a 30-G irrigation needle (Max-i-Probe; Dentsply Sirona, York, Pennsylvania, USA) up to 2 mm short of the final WL ^19^; final irrigation using 5 mL of 17% EDTA for 5 min; and neutralization with 2 mL of 0.9% saline solution.

Sterile absorbent paper cones from the Reciproc System (VDW) were used inside the root canal before obturation, which was performed with the gutta-percha cones of the system (VDW), R40 or R50, and MTA Fillapex (Angelus, Londrina, Paraná, Brazil), using lateral condensation. Finally, the tooth was restored with a definitive restorative material. The occlusion was checked and adjusted.

### Predictor Variables

Data on the following variables were collected: age, gender, arch position (maxilla or mandible), tooth length (mm), root canal amplitude (teeth instrumented with R40-medium or R50-large), and periapical lesion size (mm^2^).

The length of the teeth was obtained using the apical locator, and all teeth were standardized from the cementoenamel junction to the dental apex using the tools available on the Kodak Digital Rx (Kodak).

The amplitude of the root canals was determined by the taper of the selected Reciproc file, which was classified, according to the manufacturer, as large and medium when the Reciproc 50 and 40 files, respectively, were used for instrumentation of the root canals.

The size of the periapical lesion was determined using ImageJ/Fiji 1.46 (http://imagej.nih.gov/ij/) software. The initial standardized radiography of each case was inserted into the software, and through the delimitation of the lesion by a single operator (L.S.G) and specific tools, the lesion area was determined in mm^2^.

### Outcome Variables

The outcome variables were postoperative pain, tenderness, and edema. The use of analgesics after root canal treatment with foraminal enlargement in single-rooted teeth with periapical lesion was also noted. The analyses of postoperative pain and tenderness were performed using the visual analogue scale (VAS), which was delivered to all patients to record the pain assessment daily for 7 days and on days 14 and 30 after root canal treatment. The VAS consisted of a horizontal ruler with two endpoints (0 indicating no pain and 10 indicating severe pain). The distance from 0 to the mark made by each patient was measured with a ruler, and the resulting quantitative values were used in the statistical analysis. Pain levels were classified as no pain (0), mild ^1^ ^,^ ^2^ ^,^ ^3^, moderate ^4^ ^,^ ^5^ ^,^ ^6^, or severe ^7^ ^,^ ^8^ ^,^ ^9^ ^,^ ^10^. All patients were instructed to contact the research coordinator (L.S.A) in case of severe pain or any other complications. In cases of severe pain, the analgesic (ibuprofen 400 mg) ^19^ was prescribed every 6 hours for 5 days, according to a pre-established protocol.

Based on the criteria of Morse et al. ^20^, edema was evaluated at 48 h, 72 h, and 7 days after treatment. Two independent and blinded authors clinically evaluated edema by comparing the patient's initial photograph (k = 0.90), as follows:

1. Mild: there is no distortion of the face but slight swelling of the gingiva, cheeks, or chin.
2. Moderate: there is a superficial distortion of the cheek or chin.
3. Severe: there is a severe distortion of the part involved.

### Data Management and Analyses

The data were analyzed using the Statistical Package for Social Science software (IBM Corp, Armonk, New York, USA, version 23.0). The level of significance was set at 5% (p < 0.05).

The normality of the continuous predictor variables was evaluated using the Kolmogorov Smirnov test, as none of the variables showed a normal distribution (p<0.05). The Mann-Whitney U-test and Spearman’s rank correlation were applied to assess the association between the predictor variables and postoperative pain and tenderness daily, for 7 days, and on days 14 and 30 after root canal treatment. These predictor variables were also assessed for edema and use of analgesics using the Chi-square, Fisher’s exact and Mann-Whitney U tests. Multivariate Ordinal Regression using the Generalized Estimating Equation model (GEE) was performed to evaluate the association between VAS values and predictor variables adjusted by analgesic use. All predictor variables were added in the regression models.

## Results

**Figure 1:**
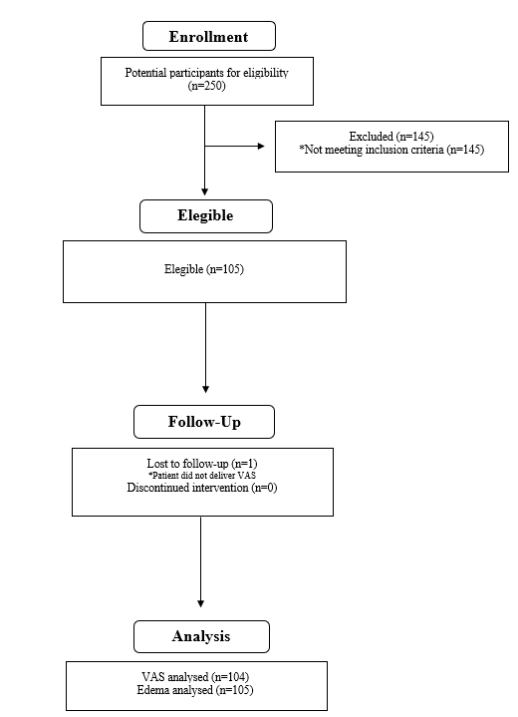
Flow diagram of the sample.

A total of 105 patients were included in this study. One patient was lost to follow-up for postoperative pain and tenderness (Figure 1). The mean age of the sample was 39.85 ± 13.00 (range 18(70) years; and 64 patients (61%) were female.

The incidences of postoperative pain and tenderness were 38.46% (n = 40) and 42.31% (n = 44), respectively. Nineteen (18.27%) patients had mild postoperative pain, 10 (9.62%) had moderate pain, and 11 (10.58%) had severe pain. Regarding tenderness, 24 (23.08%) presented with mild pain, nine (8.66%) presented moderate pain, and 11 (10.58%) presented severe pain.

Tables 1 and 2 show the analyses of the predictor variables age, gender, arch position, tooth length, root canal amplitude, and the size of the periapical lesion in relation to postoperative pain and tenderness on the analyzed days. A statistically significant difference was found in relation to gender and root canal amplitude. Postoperative pain was greater in the female participants on days 4 (p < 0.01) and 5 (p = 0.03), and in relation to tenderness, postoperative pain was greater on days 1 (p = 0.04), 2 (p = 0.01), 4 (p = 0.02), and 5 (p = 0.02). Root canals considered medium presented greater postoperative pain and tenderness in relation to the root canal considered large on days 1 (p = 0.01), 2 (p = 0.01), 3 (p < 0.01), and 5 (p = 0.05), and on days 1 (p = 0.02), 2 (p < 0.01), and 3 (p = 0.05), respectively.

Tables 3 and 4 analyzed the occurrence of edema and postoperative use of analgesic in relation to predictor variables. There was a statistically significant difference in the occurrence of edema in relation to the medium root canal amplitude (p = 0.05) (Table 3). Only 19 patients (18.1%) required medication (Table 3).

Table 5 shows the Multivariate Ordinal Regression by GEE, which evaluates the association between pain/tenderness and predictor variables. Female gender (beta = 1.02; p < 0.01), mandibular teeth (beta = 25.50; p < 0.01), medium root canal amplitude (beta = 0.93; p = 0.03), and edema (beta = 1.88; p < 0.01) were associated with increased postoperative pain, while the use of analgesics (beta = -1.82; p < 0.01) and time in days (beta = -0.23; p < 0.01) were associated with decreased postoperative pain.

Edema and the use of analgesics were also evaluated in multivariate analysis by Logistic Regression, as shown in Tables 6 and 7. Edema was associated with a risk factor for analgesic requirement (OR = 61.46; p < 0.01).

## Discussion

Postoperative signs and symptoms are undesirable for both patients and clinicians in daily endodontic practice ^2^ ^,^ ^8^. Therefore, awareness of the factors that may be associated with a higher occurrence of these signs and symptoms is necessary to predict a better prognosis for these patients. To the best of our knowledge, this is the first study to address all these variables in endodontically treated teeth with foraminal enlargement. The objective of this study was to evaluate the relationship between age, gender, arch position, tooth length, root canal amplitude, and periapical lesion size with the occurrence of postoperative signs and symptoms and use of postoperative analgesics following root canal treatment with foraminal enlargement in single-rooted teeth with periapical lesion. The null hypothesis was that there is no relationship between these factors and the occurrence of postoperative signs and symptoms and analgesic requirements. According to the results of this study, female gender, mandibular teeth, and smaller root canal amplitude had greater postoperative pain and tenderness, whereas smaller root canal amplitudes had a higher occurrence of edema. Edema were also associated with the analgesic requirements. Thus, the null hypothesis is partially rejected.

Root canal treatment with foraminal enlargement and reciprocating instrumentation in a single session was performed in 105 patients. We included only asymptomatic patients because the inclusion of teeth with preoperative pain and edema could influence the results ^7^ ^,^ ^8^. The incidence of severe postoperative pain and tenderness was considered low, similar to the findings of previous studies ^6^ ^,^ ^19^. However, endodontic pain has a wide range of reported ^2^. This disparity could be attributed to the fact that each study followed a particular protocol ^5^ and used different sample sizes and criteria when evaluating the degree of pain, edema, and other associated factors ^2^ ^,^ ^5^ ^,^ ^8^.

 The presence of microorganisms in the apical portion of the canal and even within the lesion itself has led to the proposal of cleaning, debridement, and enlargement of the apical foramen during root canal instrumentation ^19^ ^,^ ^20^ ^,^ ^21^, improving the removal of bacteria, and leading to more predictable results; and this procedure can be performed in a single session ^19^ ^,^ ^20^ ^,^ ^21^. An overview, published in 2017 ^22^, with a high level of evidence, indicated that single and multiple visits have similar repair or success rates, regardless of pulp and periapex precondition. In addition, the subgroup with periapical lesion showed a slight positive trend towards a decrease in the incidence of postoperative complications and a greater effectiveness and efficiency for a single session, stating that this clinical approach did not influence the present outcome evaluated.

The relationship between risk factors and postoperative signs and symptoms has been studied by several authors ^1^ ^,^ ^2^ ^,^ ^3^ ^,^ ^4^ ^,^ ^5^ ^,^ ^6^ ^,^ ^7^ ^,^ ^8^ ^,^ ^9^ ^,^ ^10^ ^,^ ^11^ ^,^ ^12^ ^,1,^ ^14^ ^,^ ^15^ ^,^ ^16^; however, it has not been studied in teeth instrumented with a reciprocating system with foraminal enlargement. In addition, the scientific literature does not fully address, with well-defined methodologies, the relationship of signs and symptoms with the variables tooth length, root canal amplitude, and periapical lesion size.

**Table 1:** Comparison between groups of the dichotomic predictor variables in relation to postoperative pain and tenderness per day.

	Variables	Day 1	p	Day 2	p	Day 3	p	Day 4	p	Day 5	p	Day 6	p	Day 7	p	Day 14	p	Day 30	p
Postoperative pain	Gender																		
	Women	1.30 (2.15) / 0.00	0.06	1.12 (2.13) / 0.00	0.08	1.00 (2.18) / 0.00	0.10	0.79 (1.95) / 0.00	0.00	0.50 (1.36) / 0.00	0.03	0.30 (0.92) / 0.00	0.06	0.17 (0.73) / 0.00	0.23	0.14 (0.53) / 0.00	0.25	0.06 (0.35) / 0.00	0.82
		(0.00-2.00)		(0.00-1.00)		(0.00-1.00)		(0.00-0.00)		(0.00-0.00)		(0.00-0.00)		(0.00-0.00)		(0.00-0.00)		(0.00-0.00)	
	Men	0.90 (2.18) / 0.00		0.80 (2.05) / 0.00		0.60 (1.74) / 0.00		0.04 (0.21) / 0.00		0.07 (0.34) / 0.00		0.02 (0.15) / 0.00		0.02 (0.15) / 0.00		0.14 (0.93) / 0.00		0.04 (0.31) / 0.00	
		(0.00-0.00)		(0.00-0.00)		(0.00-0.00)		(0.00-0.00)		(0.00-0.00)		(0.00-0.00)		(0.00-0.00)		(0.00-0.00)		(0.00-0.00)	
	Arch position																		
	Maxilla	1.08 (2.08) / 0.00	0.76	0.92 (2.06) / 0.00	0.46	0.91 (2.24) / 0.00	0.48	0.59 (1.81) / 0.00	0.91	0.40 (1.30) / 0.00	0.66	0.22 (0.83) / 0.00	0.85	0.16 (0.70) / 0.00	0.30	0.20 (0.87) / 0.00	0.30	0.08 (0.41) / 0.00	0.19
		(0.00-1.00)		(0.00-1.00)		(0.00-0.00)		(0.00-0.00)		(0.00-0.00)		(0.00-0.00)		(0.00-0.00)		(0.00-0.00)		(0.00-0.00)	
	Mandible	1.24 (2.32) / 0.00		1.13 (2.17) / 0.00		0.72 (1.55) / 0.00		0.32 (0.94) / 0.00		0.21 (0.58) / 0.00		0.13 (0.53) / 0.00		0.02 (0.16) / 0.00		0.02 (0.16) / 0.00		0.00 (0.00) / 0.00	
		(0.00-2.00)		(0.00-1.00)		(0.00-0.00)		(0.00-0.00)		(0.00-0.00)		(0.00-0.00)		(0.00-0.00)		(0.00-0.00)		(0.00-0.00)	
	Root canal amplitude																		
	Medium	1.57 (2.21) / 1.00	0.01	1.60 (2.19) / 0.00	0.01	1.39 (2.28) / 0.00	0.00	0.57 (1.57) / 0.00	0.18	0.46 (1.20) / 0.00	0.05	0.25 (0.96) / 0.00	0.66	0.21 (0.95) / 0.00	0.70	0.14 (0.59) / 0.00	0.72	0.07 (0.37) / 0.00	0.80
		(0.00-2.00)		(0.00-3.75)		(0.00-2.00)		(0.00-0.75)		(0.00-0.75)		(0.00-0.00)		(0.00-0.00)		(0.00-0.00)		(0.00-0.00)	
	Large	0.98 (2.13) / 0.00		0.77 (2.03) / 0.00		0.64 (1.89) / 0.00		0.47 (1.57) / 0.00		0.28 (1.06) / 0.00		0.17 (0.64) / 0.00		0.07 (0.35) / 0.00		0.14 (0.76) / 0.00		0.05 (0.32) / 0.00	
		(0.00-1.00)		(0.00-0.00)		(0.00-0.00)		(0.00-0.00)		(0.00-0.00)		(0.00-0.00)		(0.00-0.00)		(0.00-0.00)		(0.00-0.00)	
Tenderness	Gender																		
	Women	0.69 (0.90) / 0.00	0.04	0.65 (0.90) /	0.01	0.50 (0.87) / 0.00	0.13	0.39 (0.83) / 0.00	0.02	0.31 (0.69) / 0.00	0.02	0.19 (0.53) / 0.00	0.17	0.15 (0.48) / 0.00	0.25	0.12 (0.38) / 0.00	0.28	0.07 (0.32) / 0.00	0.36
		(0.00-1.00)		0.00 (0.00-1.00)		(0.00-1.00)		(0.00-0.00)		(0.00-0.00)		(0.00-0.00)		(0.00-0.00)		(0.00-0.00)		(0.00-0.00)	
	Men	0.46 (0.92) / 0.00		0.29 (0.74) / 0.00		0.29 (0.67) / 0.00		0.07 (0.26) / 0.00		0.04 (0.21) / 0.00		0.04 (0.21) / 0.00		0.04 (0.21) / 0.00		0.07 (0.34) / 0.00		0.02 (0.15) / 0.00	
		(0.00-0.50)		(0.00-0.00)		(0.00-0.00)		(0.00-0.00)		(0.00-0.00)		(0.00-0.00)		(0.00-0.00)		(0.00-0.00)		(0.00-0.00)	
	Arch position																		
	Maxilla	0.58 (0.92) / 0.00	0.56	0.43 (0.83) / 0.00	0.09	0.44 (0.87) / 0.00	0.90	0.31 (0.78) / 0.00	0.74	0.22 (0.62) / 0.00	0.79	0.14 (0.46) / 0.00	0.69	0.14 (0.46) / 0.00	0.36	0.13 (0.42) / 0.00	0.36	0.08 (0.33) / 0.00	0.09
		(0.00-1.00)		(0.00-1.00)		(0.00-1.00)		(0.00-0.00)		(0.00-0.00)		(0.00-0.00)		(0.00-0.00)		(0.00-0.00)		(0.00-0.00)	
	Mandible	0.64 (0.91) / 0.00		0.64 (0.88) / 0.00		0.37 (0.68) / 0.00		0.18 (0.46) / 0.00		0.18 (0.46) / 0.00		0.10 (0.39) / 0.00		0.05 (0.22) / 0.00		0.05 (0.22) / 0.00		0.00 (0.00) / 0.00	
		(0.00-1.00)		(0.00-1.00)		(0.00-1.00)		(0.00-0.00)		(0.00-0.00)		(0.00-0.00)		(0.00-0.00)		(0.00-0.00)		(0.00-0.00)	
	Root canal amplitude																		
	Medium	0.85 (0.93) / 1.00	0.02	0.82 (0.90) / 1.00	0.00	0.60 (0.87) / 0.00	0.05	0.35 (0.67) / 0.00	0.09	0.28 (0.53) / 0.00	0.09	0.17 (0.47) / 0.00	0.35	0.14 (0.44) / 0.00	0.65	0.10 (0.31) / 0.00	0.68	0.03 (0.18) / 0.00	0.71
		(0.00-1.00)		(0.00-1.75)		(0.00-1.00)		(0.00-1.00)		(0.00-0.75)		(0.00-0.00)		(0.00-0.00)		(0.00-0.00)		(0.00-0.00)	
	Large	0.51 (0.90) / 0.00		0.39 (0.81) / 0.00		0.35 (0.77) / 0.00		0.23 (0.69) / 0.00		0.18 (0.58) / 0.00		0.11 (0.43) / 0.00		0.10 (0.38) / 0.00		0.10 (0.38) / 0.00		0.06 (0.29) / 0.00	
		(0.00-1.00)		(0.00-0.00)		(0.00-0.00)		(0.00-0.00)		(0.00-0.00)		(0.00-0.00)		(0.00-0.00)		(0.00-0.00)		(0.00-0.00)	

Mann-Whitney U-test was performed. Bold font indicates statistical significance (p<0.05); Mean (Standard Deviation) / Median (Q1-Q3): 1^st^ and 3^rd^ quartile (25%, 75%, respectively).

**Table 2:** Correlation between continuous predictor variables in relation to postoperative pain and tenderness per day

			Age	Tooth length (mm)	Periapical lesion size (mm²)
Postoperative pain	Day 1	r	0.125	0.127	-0.060
		p	0.242	0.198	0.546
	Day 2	r	0.169	0.108	-0.005
		p	0.110	0.277	0.960
	Day 3	r	0.148	0.076	-0.010
		p	0.165	0.444	0.921
	Day 4	r	0.005	-0.064	0.004
		p	0.960	0.518	0.967
	Day 5	r	-0.005	0.042	-0.013
		p	0.965	0.671	0.898
	Day 6	r	-0.042	-0.025	0.028
		p	0.696	0.797	0.782
	Day 7	r	-0.089	-0.084	0.026
		p	0.405	0.398	0.793
	Day 14	r	-0.152	-0.076	0.093
		p	0.152	0.442	0.347
	Day 30	r	-0.128	-0.149	0.189
		p	0.230	0.130	0.054
Tenderness	Day 1	r	0.152	0.090	-0.086
		p	0.152	0.362	0.388
	Day 2	r	0.160	0.137	-0.085
		p	0.133	0.166	0.390
	Day 3	r	0.103	0.062	-0.062
		p	0.335	0.532	0.530
	Day 4	r	-0.028	-0.038	-0.061
		p	0.791	0.705	0.536
	Day 5	r	0.004	0.024	-0.045
		p	0.968	0.807	0.652
	Day 6	r	0.000	0.005	0.022
		p	0.999	0.957	0.827
	Day 7	r	-0.019	-0.061	0.012
		p	0.855	0.537	0.904
	Day 14	r	-0.089	-0.090	0.067
		p	0.402	0.362	0.501
	Day 30	r	-0.062	-0.108	0.088
		p	0.563	0.277	0.376

Spearman correlation was performed.

**Table 3:** Assessment of the occurrence of edema and postoperative use of analgesic of the dichotomic predictor variables.

Variables (n=105)		Edema (n) Yes/No	P value	Use of analgesics (n) Yes/No	P value
Gender	Women Men	6/58 1/40	0.24	14/50 5/36	0.299
Arch position**	Maxilla Mandible	3/64 4/34	0.23	13/54 6/32	0.793
Root canal amplitude**	Medium Large	4/24 3/74	0.05	7/21 12/65	0.267

* Fisher`s Exact test / **Chi-Square test: bold font indicates statistical significance (p<0.05).

**Table 4:** Assessment of the occurrence of edema and postoperative use of analgesic of the continuous predictor variables.

	Edema			Use of analgesics		
	Yes	No	p-value	Yes	No	p-value
Age	46.71 (12.08) / 47.00 (37.00 - 54.00)	39.79 (13.39) / 36.00 (31.00 - 49.50)	0.455	42.28 (12.28) / 43.00 (32.00 - 53.00)	39.84 (13.65) / 36.00 (31.00 - 49.00)	0.159
Tooth Length (mm)	12.56 (3.08) / 11.60 (10.80 - 13.00)	12.25 (2.04) / 11.80 (10.70 - 14.10)	0.848	12.56 (2.76) / 11.70 (10.70 - 14.10)	12.20 (1.95) / 11.75 (10.70 - 14.00)	0.832
Periapical lesion size (mm²)	13.17 (7.06) / 10.31 (5.75 - 20.38)	14.88 (16.12) / 9.51 (4.37 - 18.80)	0.851	14.81 (19.81) / 9.94 (5.20 - 18.03)	4.76 (14.74) / 9.51 (4.80 - 20.14)	0.537

Mann-Whitney U-test was performed. Mean (Standard Deviation) / Median (Q1-Q3): 1^st^ and 3^rd^ quartile (25%, 75%, respectively).

**Table 5:** Ordinal Regression by GEE for VAS and VAS to tenderness.

Variables	Visual Analog Scale to Pain Values				Visual Analog Scale to Tenderness Values			
	Beta	Error	CI (95%)	P	Beta	Error	CI (95%)	p
Female Gender	1.02	0.21	0.59 - 1.44	<0.001	0.98	0.19	0.59 - 1.27	<0.001
Mandibular Teeth	25.50	0.69	24.13 - 26.87	<0.001	25.83	0.68	24-48 - 27.17	<0.001
Tooth length (mm)	0.27	0.42	-1.10 - 0.55	0.514	0.35	0.43	-0.49 -1.20	0.415
Medium Root Canal Amplitude	0.93	0.45	0.47 - 1.81	0.039	0.35	0.53	-0.68 - 1.40	0.501
Periapical Lesion size (mm^2^)	0.05	0.45	-0.84 - 0.94	0.908	0.35	0.46	-0.55 - 1.26	0.446
Use of Analgesic	-1.82	0.46	-2.74 - -0.90	<0.001	-1.57	0.46	-2.49 - -0.65	0.001
Edema	1.88	0.46	0.90 - 2.74	<0.001	2.45	0.63	1.20 - 3.70	<0.001
Time in days	-0.23	0.08	0.06 - 0.41	0.007	-0.16	0.04	-0.25 - -0.07	0.001
Age	0.00	0.09	-0.00 - 0.02	0.249	-0.06	0.02	-0.04 - 0.03	0.790

CI: Confidence Interval. Bold forms mean statistical significance. Instead of the intercept, SPSS use the categories of data for the ordinal regression.

**Table 6:** Logistic Regression for Edema.

Variables	Beta	Error	OR	CI (95%)	p
Female Gender	-	-	4.13	0.48 - 35.70	0.196
Tooth Length (mm)	0.23	1.62	-	-	0.159
Large Root Canal Amplitude	-	-	0.18	0.01 - 3.32	0.250
Periapical Lesion size (mm²)	0.01	1.38	-	-	0.969
Age	0.21	0.02			0.154

CI: Confidence Interval. OR: Odds Ratio. To continuous variables, beta and error were calculated. To dichotomized or categorical variables, OR and CI were calculated. A complete or quasi-complete data separation made it impossible to analyze the arch position, and analgesic cannot be a predictor factor of edema.

**Table 7:** Logistic Regression for Postoperative Use of Analgesic.

Variables	Beta	Error	OR	CI (95%)	p
Female Gender	-	-	2.01	0.66 - 6.10	0.215
Tooth length (mm)	0.28	0.72	-	-	0.182
Large Root Canal Amplitude	-	-	0.48	0.06 - 3.49	0.475
Periapical Lesion size (mm²)	0.17	0.67	-	-	0.548
Edema	-		61.46	4.10 - 919.76	0.003
Age	0.15	0.01	-	-	0.439

CI: Confidence Interval. OR: Odds Ratio. To continuous variables, beta and error were calculated. To dichotomized or categorical variables, OR and CI were calculated. Bold forms mean statistical significance. A complete or quasi-complete data separation made it impossible to analyze arch position.

In this study, the patients’ age did not show significant effects on the occurrence of postoperative signs and symptoms. These findings aligned with several studies (^1^,^2^,^3^,^4^,^5^,^14^,^16^). However, Arias et al*.* ^12^ described a higher probability of experiencing moderate or severe pain with increasing age and mandibular teeth. The low incidence of postoperative signs and symptoms in young patients can be explained by the lack of experience and a more tolerant psychological attitude toward pain and discomfort ^8^.

Regarding the arch position, the mandibular teeth were responsible for greater postoperative pain and tenderness, which can be explained mainly because of its thick mandibular cortical plaque and the consequent lack of space for pressure dissipation due to the accumulation of inflammatory exudates ^8^.

We also observed a significant difference in the amplitude of the root canal considered as medium. This significant difference may be related to the extrusion of debris during the cleaning and shaping of the root canals. In our study, single-rooted teeth with periapical lesion were subjected to root canal treatment with reciprocating instrumentation and foraminal enlargement, and even with all well-established and evidence-based protocols ^17^ ^,^ ^21^, there is a chance of extruding debris into the periapical tissues. Apical extrusion of contaminated debris to the periradicular tissue is one of the principal causes of postoperative pain ^4^. Therefore, it is important to visualize and have knowledge of the internal anatomy before undertaking endodontic therapy ^23^.

However, it is worth pointing out that several studies ^17^ ^,^ ^21^ have demonstrated that the shaping of the root canal using reciprocating systems does not increase the apical extrusion of debris mainly because of its automated technique of balanced force without apical pressure, instrument design, improved alloy, use of fewer instruments during treatment, high cutting capacity, and reciprocation kinematics ^17^. Furthermore, the characteristics of reciprocating systems associated with microbial reduction, also provided by foraminal enlargement, encouraged the use of this technique and evaluated the influence of the factors analyzed in this study on the occurrence of postoperative signs and symptoms. In addition, 2.5% sodium hypochlorite was added to this reciprocating instrumentation. Sodium hypochlorite is the most widely used endodontic irrigator because of its pronounced antimicrobial activity and ability to dissolve organic matter. However, sodium hypochlorite can be cytotoxic to periradicular tissues, especially at high concentrations. Therefore, we carried out a specific and careful irrigation protocol using the Max-i-Probe needle ^19^, further reducing the possibility of accumulation and extrusion of debris, and consequently, the levels of postoperative pain ^4^. MTA Fillapex sealer was the material of choice for filling the root canals in this study. Although MTA Fillapex is not considered the gold standard, it has adequate physicochemical properties for an endodontic sealer ^24^. In our study, there was no intentional extrusion of endodontic sealer, thus reducing the possible confounding bias.

It is also known that a higher incidence of postoperative signs and symptoms occurs in teeth without periapical lesion or with very small lesions, which can be attributed mainly to the lack of space for pressure release when periradicular bone resorption is absent ^4^ ^,^ ^8^ ^,^ ^12^ ^,^ ^16^. However, another hypothesis may arise ^7^. Teeth with high radiolucency are more likely to develop postoperative pain due to the number and type of isolated bacterial strains ^25^. In our study, periapical lesion size was established at mm^2^ and did not show statistically significant results.

Another risk factor identified in our study as a potentially important prognostic factor for postoperative symptoms was gender, in agreement with the results obtained by the retrospective study conducted by Torabinejad et al. ^8^, who showed a significant positive correlation of symptoms in female patients. Several hypotheses have been proposed to explain this predominance. Biological differences were the most legitimate explanations. The threshold and tolerance to pain depend on sex hormones and their proportion during the different stages of the menstrual cycle. These levels may also be associated with changes in serotonin and noradrenaline levels, leading to an increased prevalence of symptoms in female patients undergoing hormone replacement therapy or using oral contraceptives and during menstrual periods. Differences in the female pelvic and reproductive organs can also provide an additional gateway for infection, leading to possible local and distant hyperalgesia. In addition, pain is also regulated by the hormone cortisol, which participates in the mechanisms responsible for pain processing, and its excretion is higher in males than in females ^6^ ^,^ ^10^ ^,^ ^15^.

Finally, the use of analgesics was directly proportional to the occurrence of postoperative signs and symptoms, thus, edema was considered a risk factor for the analgesic requirement. However, the use of postoperative analgesics and time in days was associated with decreased postoperative pain, which was already expected, as the intensity of the pain decreased substantially in the first few days. It is worth mentioning that patients were only medicated when requested, an essential factor in avoiding erroneous estimates in this study.

Although our study has a few methodological biases, it has some limitations. First, the VAS did not indicate the source of postoperative pain and tenderness. The perception of pain is subjective and strongly dependent on the patient's cultural, individual, and economic context. For this reason, the design of the questionnaire is critical and must ensure that it will be fully understood by patients and easily interpreted by researchers ^11^. The VAS is a simple and efficient model that is easy to understand and reliable for assessing pain intensity ^21^. All data were obtained without the interference of an interviewer who could influence the results. In this study, we chose to use the VAS to assess postoperative pain and tenderness, as it is widely used in the endodontic literature ^19^ ^,^ ^21^. Second, to analyze the occurrence of edema, we followed the methodology proposed by Morse et al*.* ^20^, and in this case, we used the initial photograph as a parameter. However, two independent evaluators, who did not actively participate in the procedures, performed this evaluation clinically, reducing the bias of this analysis. Third, the analysis of the periapical lesion size was obtained through two-dimensional digital radiographs, which may have led to some interference in the results. However, with the use of specific software, this bias may have been reduced.

Therefore, it is necessary to carry out other studies with adequate sample sizes and well-developed methodologies to confirm or refute the various critical factors to the postoperative signs and symptoms found in this study.

## Conclusion

Female gender, mandibular teeth, and medium root canal amplitude could be factors associated with postoperative signs and symptoms in root canal treatment with foraminal enlargement in single-rooted teeth with apical periodontitis. The use of analgesics was most required in patients who presented edema, and it was associated with decreased pain over time.
